# SARS-CoV-2 evolution enhances endocytic uptake while preserving TMPRSS2-dependent fusion

**DOI:** 10.3389/fimmu.2025.1736891

**Published:** 2026-01-12

**Authors:** Clara L. Magnus, Zeliha Hamed Jaber, Andreas Hiergeist, Lisa Arnold, Harriet Marchel, Antonia Lamprecht, Frank Hanses, Thomas Dienemann, Roland Schneckenpointner, Matthias Lubnow, Thomas Müller, Dirk Lunz, Florian Hitzenbichler, Stephan Schmid, Martina Müller, Hendrik Poeck, Bernhard Graf, Bernd Salzberger, André Gessner, Barbara Schmidt, Philipp Schuster

**Affiliations:** 1 Institute of Clinical Microbiology and Hygiene, University Hospital Regensburg, Regensburg, Germany; 2 Institute of Medical Microbiology and Hygiene, University of Regensburg, Regensburg, Germany; 3 Emergency Department, University Hospital Regensburg, Regensburg, Germany; 4 Department of Infection Prevention and Infectious Diseases, University Hospital Regensburg, Regensburg, Germany; 5 Department of Surgery, University Hospital Regensburg, Regensburg, Germany; 6 Department of Internal Medicine II, University Hospital Regensburg, Regensburg, Germany; 7 Department of Anesthesiology, University Hospital Regensburg, Regensburg, Germany; 8 Department of Internal Medicine I, University Hospital Regensburg, Regensburg, Germany; 9 Department of Department of Internal Medicine 3, University Medical Center, Regensburg, Germany; 10 Leibniz Institute for Immunotherapy, Regensburg, Germany

**Keywords:** coronavirus, endocytosis, evolution, fusion, SARS-CoV-2, tropism, variants-of-concern, viral entry

## Abstract

**Background:**

Of the five SARS-CoV-2 variants-of-concern (VOC), Omicron shows increased transmissibility and infectivity, but lower pathogenicity. This drop in virulence was associated with an altered entry of VOC Omicron into airway epithelia by endocytosis instead of direct fusion, increasing virus replication in the upper airways and decreasing spread to the lower respiratory tract.

**Methods:**

We aimed to assess the extent of direct fusion and endocytosis in nine clinical SARS-CoV-2 isolates collected during the SARS-CoV-2 pandemic, comprising wild-type (n=1), Alpha (n=2), Delta (n=1), and Omicron (n=5) strains. Viral entry was investigated in four different human cell lines in the presence of camostat, an inhibitor of TMPRSS2-mediated fusion, and aloxistatin, a cathepsin protease inhibitor blocking viral endocytic entry (0.024-100 µM). Full-length viral genomes were obtained using next generation sequencing.

**Results:**

Alpha and Delta variants predominantly entered Calu-3 and Caco-2 cells through TMPRSS2-dependent membrane fusion, whereas Omicron variants – particularly BE.1.1 and BA.5 – showed a pronounced shift toward endocytosis in A549^hACE2+/TMPRSS2+^ and HEK293T cells. Endocytic uptake was preferentially utilized by strains carrying Δ69/Δ70 and L452R in combination with F486V.

**Conclusions:**

All Omicron variants retained TMPRSS2-dependent fusion activity, indicating that VOC Omicron broadened rather than shifted its cell tropism. While replication in the upper airways and transmissibility are enhanced, the capacity to infect the lower respiratory tract is preserved, which may pose a risk for immunocompromised individuals. The combination of Δ69/Δ70, L452R, and mutations at position 486 may confer a selective advantage, as this constellation is now prevalent in nearly all circulating SARS-CoV-2 lineages.

## 1 Introduction

Since its first appearance in Wuhan, China, at the end of 2019 (1, 2), SARS-CoV-2 has spread worldwide and has since gradually adapted to the human host and its humoral and cellular immune responses (3). The original strain and in particular subsequent variants-of-concern (VOC) Alpha (B.1.1.7) and Delta (AY.33) were associated with a high morbidity and mortality (4, 5). In contrast, VOC Omicron, which occurred concurrently with mass vaccination, showed much higher infectivity with lower disease severity, at least in patients without comorbidities and without immunosuppression (6, 7).

The entry of SARS-CoV-2 into target cells requires cleavage of the spike protein into S1 and S2 subunits by furin or furin-like proteases in the virus-producing cell. The nascent virus binds to ACE-2 with its S1 receptor-binding domain (RBD). After ACE-2 engagement, the S2’ site is exposed and cleaved by transmembrane protease, serine 2 (TMPRSS2) at the cell surface or by cathepsin L within the endosome (8). After S2’ cleavage, the fusion peptide is released, leading to fusion of viral and cellular membranes at the cell surface or within the endosome, respectively, followed by the release of viral RNA into the target cell (9). During SARS-CoV-2 evolution, the mode of entry has shifted from direct TMPRSS2-mediated fusion to cathepsin L-mediated endocytosis. While the initial Wuhan strain, European wild-type (D614G), Alpha, and Delta variants entered cells mainly by direct fusion, VOC Omicron appears to favor endocytosis (9–14). This change in phenotype was associated with a lower pathogenicity of the Omicron strains in murine and hamster models (12, 13, 15, 16).

The link between the route of entry and SARS-CoV-2 pathogenicity, however, has recently been questioned. In a murine knockout model, TMPRSS2 was shown to be critical for SARS-CoV-2 entry in respiratory epithelia, including Omicron (17). In a similar model, TMPRSS2 was required for the spread of VOCs Beta and Omicron in the upper and lower respiratory tract (18). These data are supported by results in human respiratory and intestinal organoids, where infections with Omicron strains BA.1 and XBB1.5 were also dependent on TMPRSS2 (19). In addition, peptide-based pan-coronavirus fusion inhibitors potently blocked the entry of XBB and XBB.1.5 strains into lung-derived Calu-3 cells (20).

One explanation for this discrepancy may be that the usage of TMPRSS2 is different for the various Omicron subvariants. For example, BA.5 was recently shown to have a higher replication capacity and infectivity in nasal organoids than BA.1, associated with prominent syncytium formation and more efficient usage of TMPRSS2 (21). On the other hand, the infectivity and transmissibility of SARS-CoV-2 may be only partially reflected by the interaction of the viral spike protein with the cellular receptors for entry. In addition, a role of non-structural proteins (NSP) 6 and 12 in SARS-CoV-2 virulence has recently been described (22, 23).

Our goal was to elucidate how SARS-CoV-2 entry mechanisms evolved during the pandemic. Using nine clinical isolates, including five Omicron strains collected at different time points, we investigated viral entry in four human cell lines in the presence of an inhibitor of TMPRSS2-mediated fusion and a cathepsin protease inhibitor blocking viral endocytic entry. We focused on variant-specific amino-acid substitutions that may alter entry pathways and contribute to SARS-CoV-2 pathogenesis.

## 2 Materials and methods

### 2.1 Cell culture

SARS-CoV-2 strains were propagated in human Calu-3, Caco-2 (CLS Cell Lines Service, Eppelheim, Germany), A549^hACE2+/TMPRSS2+^ (InvivoGen, San Diego, US), and HEK293T cell lines using DMEM (Gibco, Waltham, MA) supplemented with 1% streptomycin/penicillin and 10% fetal calf serum (Pan Biotech, Aidenbach, Germany). Calu-3 and Caco-2 cells support SARS-CoV-2 cell entry mainly via ACE-2/TMPRSS2-mediated direct fusion (9, 24, 25), while cathepsin L-mediated endocytosis is preferentially used in A549^hACE2+/TMPRSS2+^ and HEK293T cells (24, 26, 27). Cells were split twice weekly and tested quarterly for mycoplasma contamination. The day before infection, cells were plated at a concentration of 15.000 cells/well in 96-well-plates.

### 2.2 Viruses

European wild-type strain CJ (B.1.1, GenBank accession no. PP125281) was isolated during the first SARS-CoV-2 wave in Germany (April 2020). VOC Alpha (B.1.1.7 Q27*K68*, PP125282, January 2021; B.1.1.7 Q27*, PP125283, February 2021) and Delta isolates (originally designated B.1.617.2, OK149285, June 2021; now AY.33 according to the most recent classification) were obtained during the second and third SARS-CoV-2 wave (28), respectively. Five VOC Omicron strains (BA.1.17.2, PP125291, January 2022; BA.1.1, PP125284, February 2022; BA.2.9, PP125292, March 2022; BE.1.1, PP125294, June 2022; BA.5.1, PP125293, June 2022) were isolated subsequently. The collection of patients’ respiratory samples was approved by the Ethical Commission at the Faculty for Medicine, University of Regensburg (COVUR study, Ref. no. 20-1785–101 from April 9, 2020).

Viruses were isolated on different cell lines (see above) using 1% amphotericin B (PAN Biotech, Aidenbach, DE) and 0.01% vancomycin (Hikma Pharmaceuticals, London, UK) to prevent fungal and bacterial infection. Viral replication was monitored using RT-qPCR (see below) for two to seven days post infection. Viral supernatants were harvested at the suspected peak of viral replication, purified through 0.22μm pore size filters (Carl Roth GmbH, Karlsruhe, DE), and frozen in aliquots at -80 °C. Cell culture experiments were performed under biosafety level 3 conditions according to regulatory requirements. The TCID_50_ was determined using limiting dilution in 96-well-plates according to the method of Reed and Munch (1938).

### 2.3 Inhibitors

Aloxistatin and camostat (both MedChemExpress, Monmouth Junction, NJ) were used at concentrations of 0.024-100 µM to inhibit SARS-CoV-2 endocytic uptake and direct TMPRSS2-mediated fusion on the cell surface, respectively. Inhibitors were used as single agents or in combination as a 1:1 mixture. The toxicity of both drugs at the indicated concentrations was determined using the 3-(4,5-dimethylthiazol-2-yl)-2,5-diphenyl-tetrazolium bromide (MTT) assay (**Supplementary Figure S1**), as described previously (29). Cells were preincubated with the inhibitors for 1 hour before infection, using an MOI of 0.015 for each virus isolate. To reduce the input virus, cell culture supernatants were replaced with fresh medium containing the respective inhibitor concentrations at 12–16 hours post infection (p.i.), without performing additional washing steps. This procedure resulted in an average reduction of the viral background by two to three orders of magnitude (**Supplementary Figure S2**). Final viral loads were determined two days p.i.. Viral strains with less than 0.5 log_10_ RNA copies/ml above background control in the absence of inhibitors were classified as non-replicative in this cell line. The experiments were conducted in at least three completely independent runs, and results are presented as mean values with the standard error.

### 2.4 Determination of viral load

Cell culture supernatants were mixed with an equal amount of DLR buffer (0.1 M NaCl, 0.01 M Tris, 0.5% IGEPAL CA-630 in DEPC H_2_0, pH 7.4) plus RNAse inhibitor (Applied Biosystems, Darmstadt, Germany) (30). After 30 min, RT-qPCR was performed using a published protocol on a StepOnePlus Real-time PCR system (31). *In vitro* transcribed RNA served as reference for quantification (32). Controls included cells without inhibitor and without virus (“cell control”), cells without inhibitor and with virus (“virus control, VC”), and cells fixed with 4% PFA (Sigma-Aldrich, St. Louis, MO) (“background control, BC”). The latter were washed five times with DPBS and infected in parallel on separate plates to prevent evaporation and interference with non-fixed cell layers and viruses.

### 2.5 Next generation sequencing

NGS was performed with RNA extracted from respiratory specimen using the EZ1 Advanced XL platform (Qiagen, Hilden, Germany) (29). Viral load was quantified using the real-time PCR described above. After normalizing viral copy numbers, SARS-CoV-2 RNA was reverse-transcribed using the IonTorrent™ NGS Reverse Transcription Kit (Thermo Fisher Scientific, Waltham, USA) and amplified in 247 separate amplicons using the Ion AmpliSeq™ SARS-CoV-2 Insight Research Assay (Thermo Fisher Scientific, Waltham, USA). The sequencing libraries were automatically prepared by the IonChef™ instrument (Thermo Fisher Scientific) and quantified on a LightCycler 480 II instrument (Roche Diagnostics, Mannheim, Germany) using the Ion Library TaqMan™ Quantitation Kit. After high-throughput sequencing on the IonTorrent™ Genestudio S5 Plus instrument (Thermo Fisher Scientific), basecalling and demultiplexing were performed using the Torrent Suite 5,18. Processed reads were further analyzed using the SARS-CoV-2 Research Plug-in Package and aligned to the SARS-CoV-2 MN908947 (Wuhan-Hu-1) reference genome. Consensus sequences using the generateConsensus v5.16.0.10 were generated applying a minimum read depth of 20 and a maximum of 5 percent ambiguous bases. Single nucleotide polymorphisms, detected by variantCaller v5.18.0, were annotated with COVID19AnnotateSnpEff v5.16.0.5.

For isolates Omicron (BA.1.1), Omicron (BA.1.17.2), Omicron (BE.1.1), and Omicron (BA.5.1), gaps of 30–50 N-bases with a sequencing depth below the cutoff were filled by re-sequencing using the Midnight Expansion Kit, which generates overlapping 1,200 bp PCR products. The resulting amplicon libraries were sequenced on a MinION Mk1B instrument using the Rapid Barcoding Kit V14 and an R10.4.1 flow cell (Oxford Nanopore Technologies, Oxford, UK). Consensus sequences were generated using the epi2me-labs wf-artic nextflow command line workflow with default parameters.

SARS-CoV-2 isolates were assigned to the PANGO designation based on the SARS-CoV-2 mutation analysis (pangolin-data v1.23.1, release October 27, 2023) of the Stanford Coronavirus Antiviral & Resistance Database (https://covdb.stanford.edu/sierra/sars2/by-patterns/, accessed on January 11, 2024) (33). Amino acids were numbered according to the GISAID CoV mutation App (https://gisaid.org/).

### 2.6 Phylogeny and statistics

The phylogenetic relationship of the nine virus strains was determined by analyzing the full-length nucleotide sequences using the open-source platform Nextclade v3.1.0 (https://clades.nextstrain.org/). The contribution of cathepsin-mediated endocytosis and TMPRSS2-mediated fusion to viral entry was expressed as percentage of the inhibition by the combined blockade with camostat and aloxistatin based on the viral load (VL) determined at the non-toxic inhibitor concentration of 25 µM using the following equation: $\frac{\text{log}10\text{ VLVC }–\text{ log}10\text{ VLinhibitor}}{\text{log}10\text{ VLVC }–\text{ log}10\text{ VLmixture}}$, where inhibitor means aloxistatin or camostat, respectively, and “mixture” means a 1:1 ratio of both inhibitors. Statistics was performed using two-way ANOVA with Šidák’s correction to account for multiple comparisons.

## 3 Results

### 3.1 Isolation of different SARS-CoV-2 strains in the course of the pandemic

**Figure 1:**
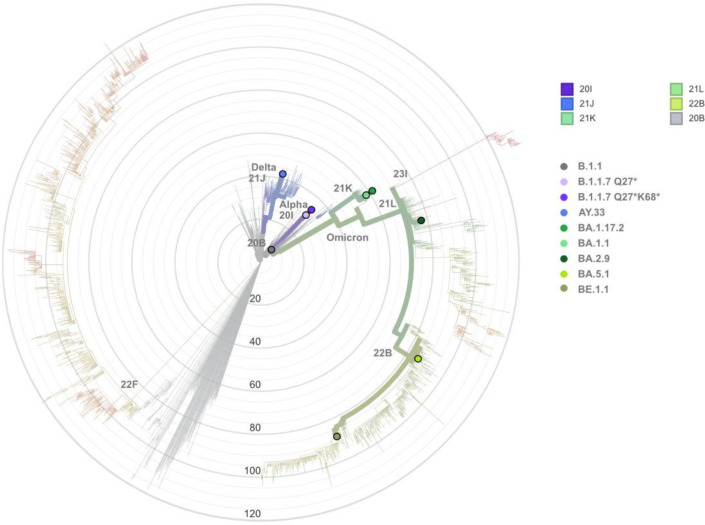
Phylogenetic analysis of the nine SARS-CoV-2 strains of our study using the open-source platform Nextclade (https://clades.nextstrain.org/). The individual strains (European wild-type B.1.1/20B (black), Alpha variants B.1.1.7 Q27*/20I (light purple) and B.1.1.7 Q27*K68*/20I (dark purple), Delta variant AY.33/21J (blue), as well as Omicron strains BA.1.17.2/21K, BA.1.1/21K, BA.2.9/21L, BE.1.1/22B, and BA.5.1/22B (green color panel), designated to PANGO lineages and Nextstrain clades, respectively, are incorporated into a radial phylogenetic reference tree and are identified by colored dots. Clades 22F and 23I include SARS-CoV-2 strains XBB and BA.2.86/JN.1., respectively. The radius of the circles reflects the number of mutations between the SARS-CoV-2 variants. The phylogenetic relationships between the strains are shown as bold branches.

From April 2020 until June 2022, we isolated nine different SARS-CoV-2 strains out of 86 respiratory samples of patients being treated at the University Hospital Regensburg. Phylogenetic analysis using the platform Nextclade revealed that the nine strains of our study, which reflected the course of the pandemic, clustered with their respective clades (**Figure 1**).

### 3.2 Omicron strains are more susceptible to blockers of cathepsin-mediated endocytosis than Alpha and Delta strains

**Figure 2:**
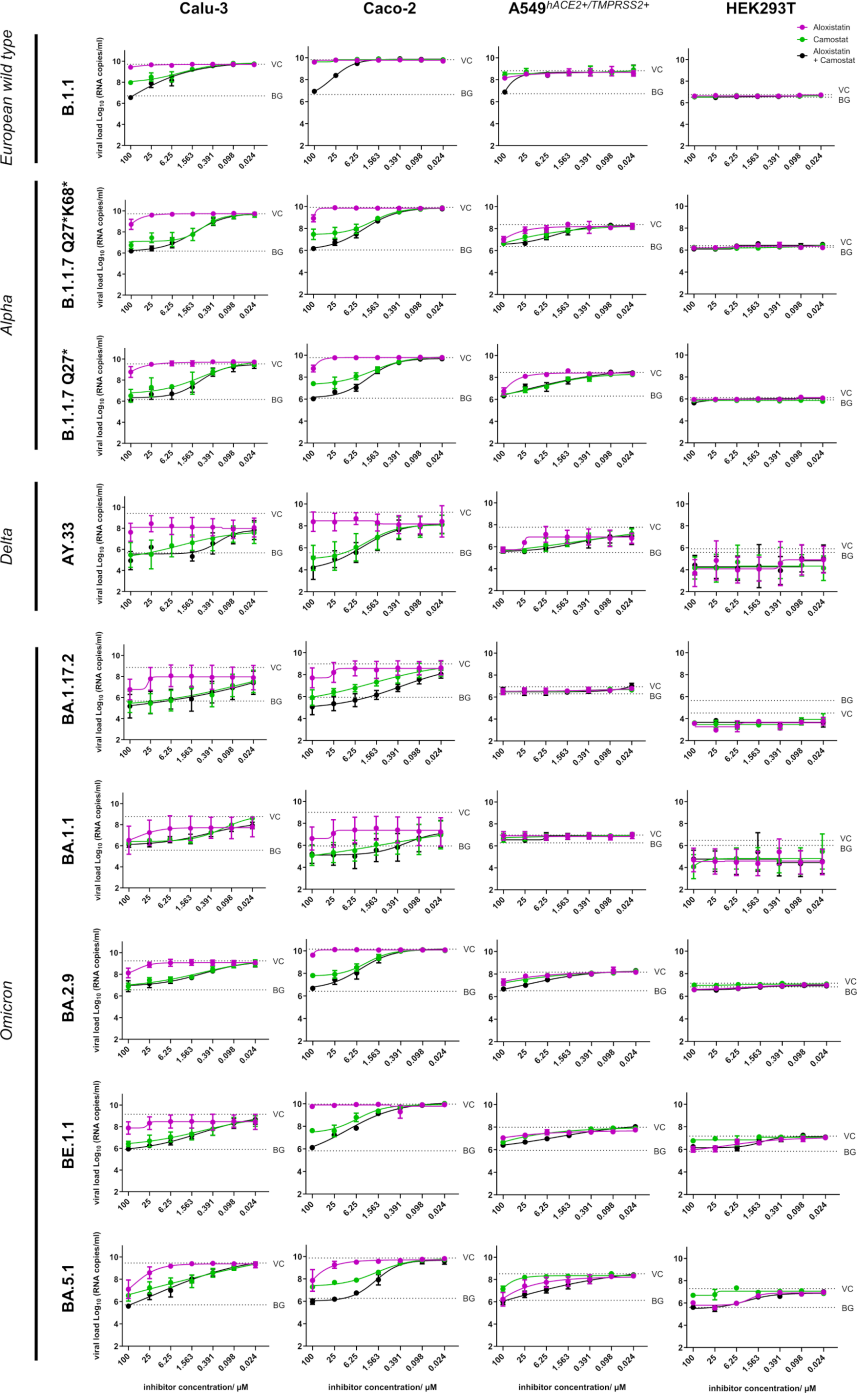
Susceptibility of the nine different SARS-CoV-2 strains to inhibitors of TMPRSS2-mediated fusion and cathepsin-mediated endocytosis. SARS-CoV-2 European wild-type (B.1.1), variant-of-concern (VOC) Alpha (B1.1.7 Q27*K68*, B.1.1.7 Q27*), Delta (AY.33), and Omicron (BA.1.17.2, BA.1.1, BA.2.9, BE.1.1, BA.5.1) were propagated in four human cell lines (Calu-3, Caco-2, A549^hACE2+/TMPRSS2+^, HEK293T) in the presence of increasing concentrations (0.024-100 µM) of camostat, an inhibitor of TMPRSS2-mediated viral direct fusion with cellular membrane (green curve), aloxistatin, an inhibitor of cathepsin-mediated viral endocytic uptake (pink curve), and a 1:1 mixture of both inhibitors (black curve). Viral load was determined in cell culture supernatants 48h p.i. using RT-qPCR and are presented as log_10_ RNA copies/ml. Controls included cells infected with SARS-CoV-2 in the absence of inhibitors (“virus control”, VC), and cells fixed with 4% paraformaldehyde and exposed to SARS-CoV-2 (“background control”, BG), shown as dashed horizontal lines. Data show mean and standard error of three independent experiments.

The route of entry of the nine viral strains was studied using four different cell lines. The inhibitors aloxistatin and camostat were non-toxic as single agents or in combination in all four cell lines up to a concentration of 25 µM (**Supplementary Figure S1**). In the absence of inhibitors, all strains showed an efficient replication in Calu-3 and Caco-2 cells with viral loads ranging from 10^9^–10^10^ RNA copies/ml within 48 hours p.i. (**Figure 2**). The combination of both inhibitors reduced viral replication of all strains to background level. The two Alpha strains, which were genetically identical except for one mutation in ORF3, behaved similarly. Aloxistatin was less effective than the mixture in blocking European wild-type, Alpha, Delta, and Omicron strains in most assays. In addition, aloxistatin inhibited BA.1.17.2, BA.2.9 and BE.1.1 entry in Calu-3 cells less effectively than camostat, indicating that the reduction in viral load by camostat, resulting from its inhibition of TMPRSS2-mediated fusion, was also more pronounced in the Omicron strains.

In A459^hACE2+/TMPRSS2+^ cells, SARS-CoV-2 strains replicated with viral loads ranging from 10^6^–10^9^ RNA copies/ml (**Figure 2**). The early (BA.1.17.2, BA.1.1) in contrast to the later Omicron strains (BA.2.9, BE.1.1, BA.5.1) were unable to replicate in this cell line. Alpha and Delta strains were blocked more strongly by camostat, whereas both inhibitors were similarly active against Omicron strains BA.2.9 and BE.1.1. BA.5.1 was preferentially blocked by aloxistatin. The mixture of both inhibitors resembled the inhibitory effect of camostat in Alpha and Delta strains, but was more effective than the individual inhibitors in Omicron strains.

**Figure 3:**
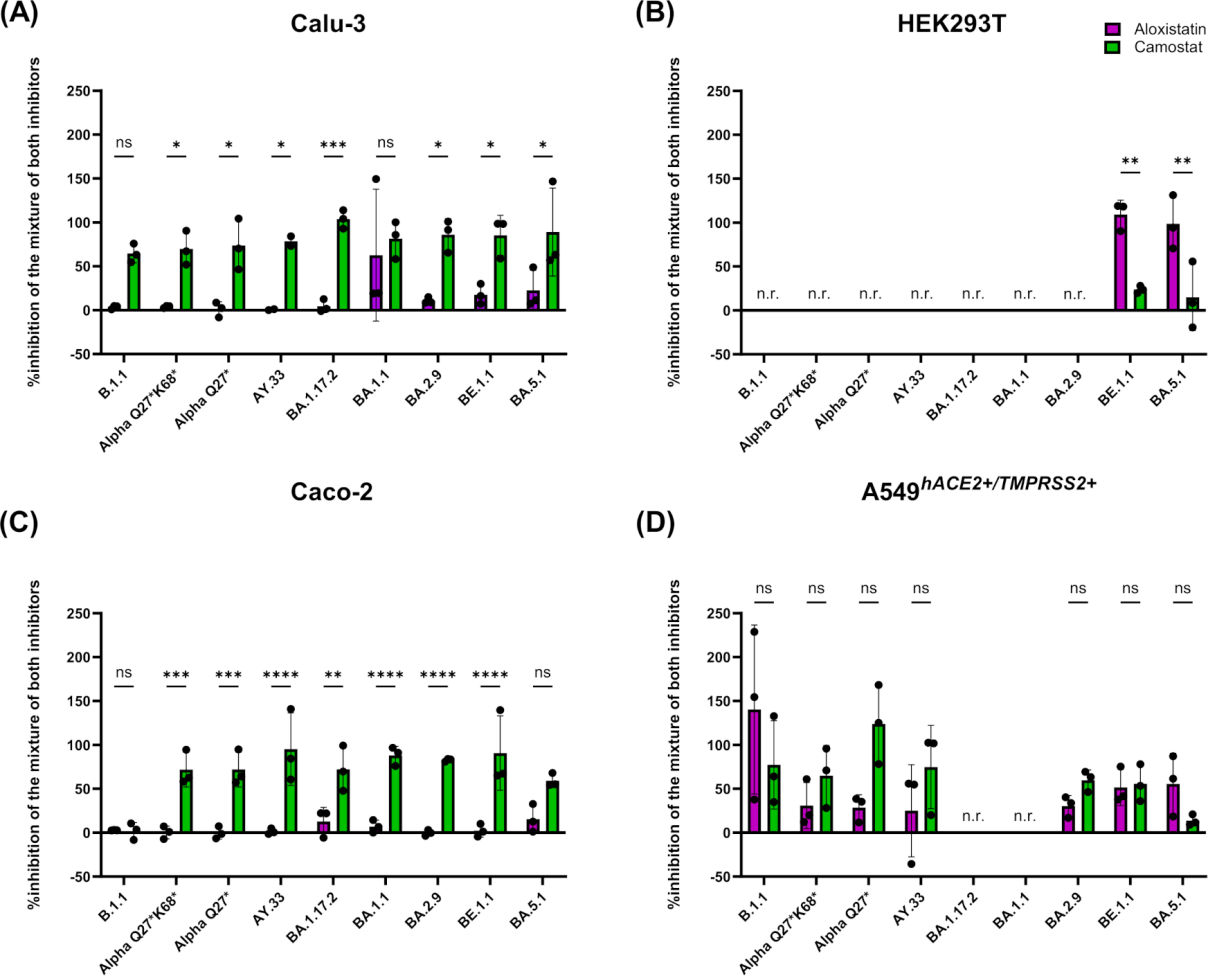
Degree of direct TMRPSS2-mediated fusion and cathepsin-mediated endocytic uptake of the nine different SARS-CoV-2 strains in four different cell lines. Calculation of the reduction in viral load induced by aloxistatin (pink columns) and camostat (green columns) at the non-toxic concentration of 25 µM as % inhibition induced by the mixture of the two inhibitors for Calu-3 cells **(A)**, HEK293T cells **(B)**, Caco-2 cells **(C)** and A549^hACE2+/TMPRSS2+^ cells **(D)**. A higher percentage indicates a stronger role of the respective mode of entry for the respective virus strain. In A549^hACE2+/TMPRSS2+^ cells, strains BA.1.17.2 and BA.1.1 were non-replicative (n.r.). In HEK293T cells, virus replication was observed for strains BE.1.1 and BA.5.1 only. Statistics was performed using two-way ANOVA with Šidák’s correction to account for multiple comparisons. *p<0.05, **p<0.01, ***p<0.001, ****p<0.0001.

In HEK293T cells, which predominantly support endocytic entry, only two variants (BE1.1, BA.5.1) showed substantial replication and were inhibited more effectively by aloxistatin than by camostat (**Figures 2**, **3B**). The mixture of both inhibitors was just as active as aloxistatin alone. When summarizing the results in all cell lines, the two most recent Omicron strains in our panel were more sensitive to inhibition of cathepsin-mediated endocytosis than the other strains, but still sensitive to inhibition of direct TMPRSS2-mediated fusion.

### 3.3 Increase and decrease in SARS-CoV-2 TMPRSS2-mediated entry during the course of the pandemic

In order to directly compare the degree of cathepsin-mediated endocytosis or TMPRSS2-mediated fusion of all virus strains, we calculated the reduction in viral load by aloxistatin or camostat at 25 µM as the percentage of the inhibition by the combined inhibitor blockade (**Figure 3**). In Calu-3 and Caco-2 cells, all strains except for two were inhibited significantly better by camostat than by aloxistatin (p<0.05, **Figure 3A**, and p<0.01, **Figure 3C**), whereas the opposite was the case for the replicative strains BE.1.1 and BA.5.1 in HEK293T cells (p<0.01) (**Figure 3B**). In A549^hACE2+/TMPRSS2+^ cells, camostat blocked Alpha Q27* and Delta AY.33 more efficiently than aloxistatin, while two of the three replicating Omicron variants responded similarly to both inhibitors; BA.5.1, in particular, showed enhanced endocytic entry reflected by stronger inhibition by aloxistatin (**Figure 3D**).

Overall, the results showed a trend in which entry of clinical SARS-CoV-2 isolates via TMPRSS2-mediated membrane fusion into Calu-3 and Caco-2 cell lines remained relatively constant over the course of the pandemic. In contrast, entry via endocytosis appeared to increase with the emergence of VOC Omicron.

### 3.4 Endocytic uptake preferentially utilized by strains carrying Δ69/Δ70, L452R, and F486V

**Figure 4:**
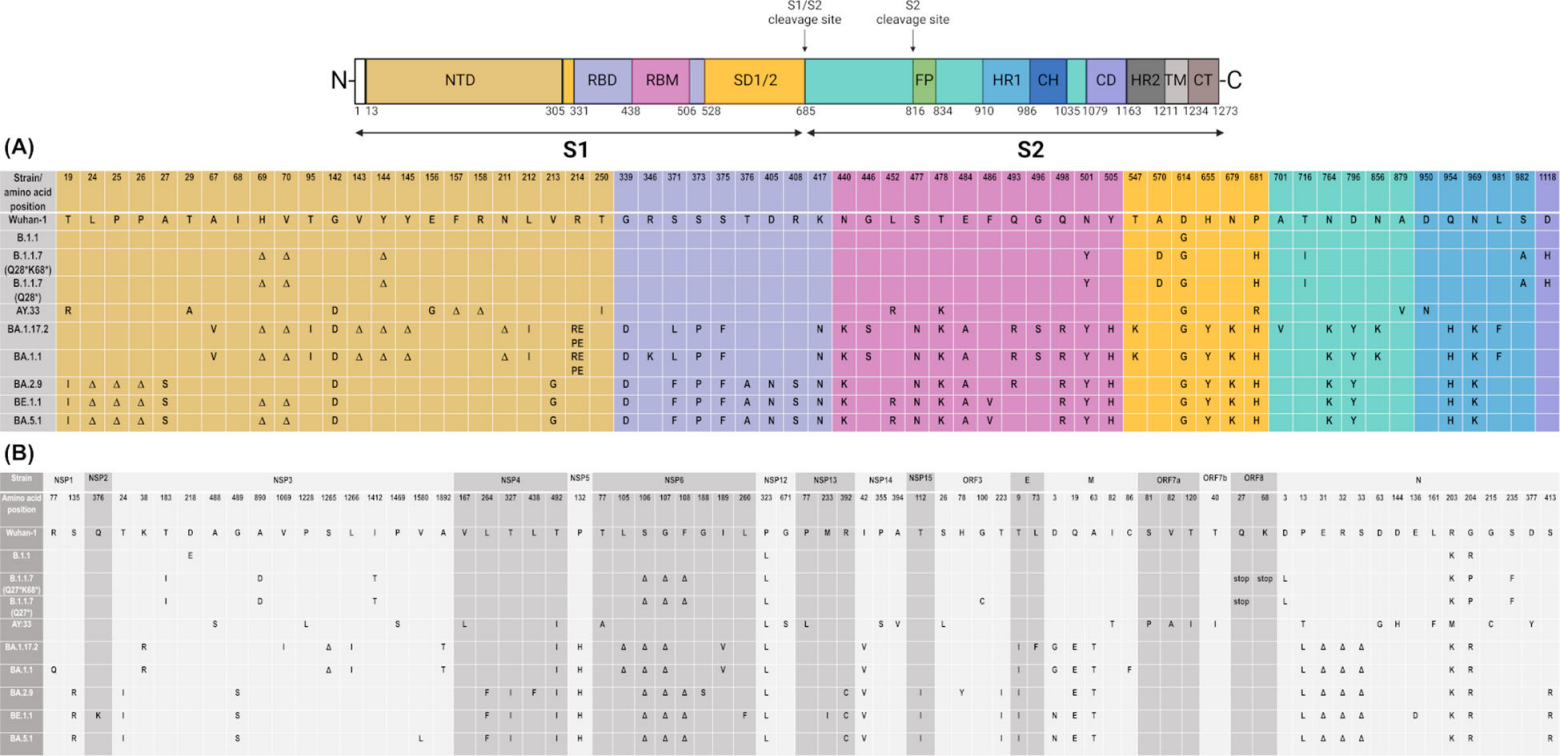
Amino acid substitutions within the viral spike protein **(A)** and in non-spike viral proteins **(B)** of the nine SARS-CoV-2 isolates. The schematic composition of the SARS-CoV-2 spike (S) protein shown above was adapted from Duan et al. (2020) under the terms of the Creative Commons Attribution License (CC BY) (49). **(A)** The amino acid differences in the S1 and S2 domain based on next generation sequencing are shown relative to the Wuhan-Hu-1 reference genome (MN90847). Colors correspond to the S-protein domain structure shown above. Δ stands for amino acid deletions, REPE for amino acid insertions at the respective position. **(B)** Amino acid substitution across the viral non-structural proteins (nsps), the envelope (E) protein, the membrane (M) protein, the nucleocapsid (N) protein, and the accessory open reading frames (ORFs) are likewise shown relative to Wuhan-Hu-1 reference. Amino acid positions follow the GISAID CoV Mutation App (https://gisaid.org/) and are labelled with one-letter-codes for **(A, B)**. To differentiate between the two Alpha strains, the second stop mutation in ORF8 of isolate B.1.1.7 Q27*K68* is included. A number of genes (NSP7, NSP8, NSP9, NSP10, NSP11, NSP16, ORF6) showed no mutations and are therefore not listed. Abbreviations according to the arrangement in the viral genome: NTD, N-terminal domain; RBD, receptor-binding domain; RBM, receptor-binding motif; SD, spike domain; FP, fusion protein; HR, heptad repeat; CH, central helix; CD, connector domain; TM, transmembrane domain; CT, cytoplasmic tail. ‘stop’ represents a stop mutation.

To correlate the mode of entry of the nine SARS-CoV-2 strains with the amino acid sequence in the viral spike protein, we analyzed the patients’ virus isolates using NGS. The wild-type strain of our study carried D614G in the spike protein as described for European strains at the beginning of the SARS-CoV-2 pandemic (34). Likewise, Alpha and Delta strains carried the prototypical amino acid exchanges, namely Δ69, Δ70, Δ144, N501Y, A570D, D614G, T716I, S982A, D1118H and T19R, G142D, Δ156, Δ157, R158G, L452R, T478K, D614G, P681R, respectively (3). Instead of P681R, the prototype mutation found in Delta strains, the two Alpha strains, like the Omicron strains, carried P681H. The Delta strain carried T250I, A879V, and D950N in addition to T29A, which are absent in Omicron strains (**Figure 4A**).

Omicron strains are characterized by a large number of amino acid exchanges in the viral spike protein (35), which were also detected in our isolates. The two earlier strains (BA.1.17.2, BA.1.1) carried A67V, Δ69, Δ70, T95I, G142D, Δ143, Δ144, Δ145, Δ211, L212I, G339D, S371L, S373P, S375F, K417N, N440K, G446S, S477N, T478K, E484A, Q493R, G496S, Q498R, N501Y, Y505H, T547K, D614G, H655Y, N679K, P681H, N764K, D796Y, N856K, Q954H, N969K, L981F and a four-amino acid insertion (REPE) at position 214. Their mutation profiles were very similar and differed from those of the two most recent strains.

BE.1.1 and BA.5.1 carried T19I, Δ24, Δ25, Δ26, A27S, Δ69, Δ70, G142D, V213G, G339D, S371F, S373P, S375F, T376A, D405N, R408S, K417N, N440K, L452R, S477N, T478K, E484A, F486V, Q498R, N501Y, Y505H, D614G, H655Y, N679K, P681H, N764K, D796Y, Q954H, N969K and wild-type amino acids at positions 67, 95, 143, 144, 145, 211, 212, 214, 446, 493, 496, 547, 856, and 981. Strain BA.2.9 closely resembled these strains, the only differences being Q493R and wild-type amino acids at positions 69, 70, 452, and 486.

Strains BE.1.1 and BA.5.1 showed increased sensitivity to the endocytosis inhibitor aloxistatin, most evident in HEK293T cells (p<0.01, **Figure 3B**), while BA.2.9 was more similar to the camostat-sensitive phenotype of the two earlier Omicron isolates (BA.1.17.2, BA.1.1) in Calu-3 and Caco-2 cells. In the Omicron strains of our study that exhibited an aloxistatin-sensitive phenotype and replicated in HEK293T cells, the Δ69/Δ70 deletion and L452R substitution, characteristic of the fusogenic Alpha and Delta variants, respectively, co-occurred with F486V.

### 3.5 Accumulation of amino acid substitutions outside the spike protein

Recent data show that the virulence of SARS-CoV-2 is not only determined by the viral spike protein, but also by changes in non-structural proteins (NSP) 6 and 12. Amino acid substitutions in these genes apparently contribute to the improved transmissibility and lower pathogenicity of the Omicron strains (22, 23). To investigate the occurrence of such changes in more detail, we analyzed the sequence information outside the viral spike protein for our nine SARS-CoV-2 strains.

Over the course of the pandemic, the number of amino acid substitutions increased in 17 SARS-CoV-2 NSPs (**Figure 4B**), while seven (NSP7, NSP8, NSP9, NSP10, NSP11, NSP16, and ORF6) remained unaffected. Amino acid exchanges that occurred in at least three viral strains were localized in NSP1 (S135R), NSP3 (T24I, G489S), NSP4 (L264F, T327I, T492I), NSP5 (P132H), NSP6 (Δ106, Δ107, Δ108), NSP12 (P323L), NSP13 (R392C), NSP14 (I42V), NSP15 (T112I), ORF3 (T223I) and in structural proteins E (T9I), M (A62T, Q219E), and N (P13L, Δ31-33, R203, G204R, S413R).

Most of these amino acid substitutions occur in the context of Omicron evolution. Amongst them, Δ106–108 and I189V in NSP6 (22) and P323L/G671S or P323L in NSP12 (23) were associated with increased SARS-CoV-2 replication in the upper respiratory tract. In our data set, all strains harbored P323L in NSP12, and therefore the differences in entry observed among the Omicron strains cannot be attributed to this substitution. Deletions in NSP6 were observed in the two earlier (Δ106-107) and in the three most recent Omicron strains (Δ106-108), but did not discriminate between more TMPRSS2-dependent (BA.1.17.2, BA.1.1, BA.2.9) and more cathepsin-dependent strains (BA.5.1, BE.1.1). More notable was amino acid substitution D3N in the M protein, a prototypic mutation for BA.4/BA.5 strains (36), which was also observed in the endocytotic BE.1.1 and BA.5.1 strains of our study, while the more TMPRSS2-dependent isolates carried D or G at this position.

## 4 Discussion

Our study used nine clinical SARS-CoV-2 strains obtained over the course of the pandemic to investigate how viral entry mechanisms evolved over time. Other groups used pseudotyped virions (37), which are perfectly suited to compare the effect of different spike proteins and distinct amino acid substitutions on entry. Pseudotyped viruses, however, do not complete the viral life cycle and do not reflect the effect of genes outside the spike protein. Therefore, we have obtained SARS-CoV-2 wild-type, Alpha and Delta isolates, and five additional strains from the difficult-to-isolate VOC Omicron (10, 11, 16). These strains enabled us to analyze the susceptibility to fusion inhibitor camostat and endocytosis blocker aloxistatin using live virus assays, which better reflect virus biology than single-replication assays. However, there are limitations to this approach, as tests with replicative viruses cannot distinguish between the role of entry and replication, which may lead to confounding effects. In addition, efficient isolation in cell culture may result in the selection of particularly infectious viral strains that do not adequately reflect the majority of circulating viruses. Furthermore, primary virus isolates may not be equally replicative in different cell lines, as we observed for BA.1.1 carrying spike mutation R346K, which showed reduced fitness in Calu-3 cells. This phenomenon was also observed by others (13) and could explain the variable infectivity of this strain in Calu-3 cells.

In the two cell lines with high-level expression of fusion-associated TMPRSS2 (Calu-3, Caco-2) (9, 24, 25), wild-type, Alpha, Delta and most Omicron strains were efficiently blocked by camostat but not by aloxistatin. These results confirm data by others showing that TMPRSS2 is not only used by early SARS-CoV-2 VOCs, but is also required for the entry of pseudotyped Omicron strains into Calu-3 cells *in vitro* (14, 19). In contrast, other studies report that the entry of Omicron into Calu-3 occurs predominantly through endocytosis and that direct TMPRSS2-mediated fusion plays a particularly important role *in vivo* (17). The inhibitory effect of aloxistatin was more pronounced in the cell lines supporting endosomal uptake of SARS-CoV-2 (A549^hACE2+/TMPRSS2+^, HEK293T) (24, 26). While Alpha and Delta strains were predominantly dependent on direct fusion in A549^hACE2+/TMPRSS2+^ cells, we observed an increasing sensitivity to aloxistatin in the two most recent Omicron BE.1.1 and BA.5.1 strains, consistent with recent findings by Willett et al. (14). For these strains, we noticed an effective utilization of the cathepsin-dependent entry pathway in HEK293T cells. In conclusion, all SARS-CoV-2 strains analyzed in our study retained the ability for direct fusion, and Omicron isolates were additionally capable to enter via endocytosis. Thus, SARS-CoV-2 appears to have expanded rather than shifted its cell tropism over the course of the pandemic.

Notably, our five Omicron sublineages differed in viral entry, which is explained by the large genetic variability between these isolates. Accordingly, sequence analyses have shown that these viruses are grouped in different branches of the phylogenetic tree (**Figure 1**). This evolution has been linked to immunological pressure from large-scale infection and/or vaccination that drove the diversification of SARS-CoV-2 spike proteins (38). By generating spike chimeras, Strobelt and colleagues showed that Omicron’s F375 reduced infectivity, and Omicron’s Y655, K764, K856, and K969 amino acid exchanges decreased TMPRSS2 dependency and supported endosomal entry (37). Similar data were reported by Qu et al., who demonstrated K547 and Y655 amino acid substitutions to be responsible for the low fusogenicity and enhanced endosomal uptake of Omicron (39). Khatri et al. associated the amino acid substitution H681 in Omicron (as opposed to R681 in Delta) with a lower fusion rate (40). The Omicron strains in our study carried all (BA.1.17.2, BA.1.1) or five of these substitutions (BA.2.9, BE.1.1, BA.5.1), but this did not explain the differences in TMPRSS2-mediated fusion and cathepsin-mediated endocytosis within our Omicron strains.

When correlating the mode of entry with the amino acid sequence of the viral spike protein, F486V (in the context of Δ69/Δ70 and L452R) was the only amino acid exchange in the two virus isolates (BE.1.1, BA.5.1) that were able to replicate in HEK293T cells and showed stronger inhibition by aloxistatin than the other VOCs. F486V was described as an escape mutation in response to neutralizing antibodies triggered by immune responses to Omicron subvariants or Omicron-based vaccine booster (41, 42). In conjunction with the Delta marker mutation L452R, F486V appears to contribute to the enhanced replicative fitness and syncytium formation of Omicron BA.5 in nasal airway organoids (21). In the pseudotype model, the combination of Δ69/Δ70, L452R, and F486V introduced by targeted mutagenesis contributed to reduced infectivity in Caco-2 cells, but enhanced infectivity in A549^hACE2+^ and in A549^hACE2+/TMPRSS2+^ cells (43). This phenotype may, at least in part, be explained by an increased propensity of this strain to use the endocytic entry pathway, while TMPRSS2-dependent entry remains preserved.

Recently, the WHO classified JN.1 (‘Juno’) as a variant of interest (VOI) and XFG as a variant under monitoring (VUM). Both lineages are now widespread across multiple countries and show extensive diversification into sublineages, while harboring a remarkably high number of Spike mutations (44). Among these are the combinations Δ69/Δ70 and L452R with F486S in XBB.1, F486P in XBB.1.6 and JN.1, and F486P/N487D in XFG. Notably, the epidemiological history demonstrates that this specific mutation pattern, identified in BE.1.1 and BA.5.1 in 2022, has since become dominant in currently circulating variants, supporting the notion that expansion of the endocytic entry pathway confers a selective advantage in terms of enhanced transmissibility.

To date, available data do not indicate that these variants are associated with increased morbidity or mortality in infected individuals. This is most likely due to the widespread development of robust immunity within the population as a result of vaccination or prior infection. Nevertheless, careful monitoring—particularly in immunocompromised patients—remains warranted, since these variants still retain the capacity for TMPRSS2-dependent infection and may therefore cause severe disease of the lower respiratory tract.

The pathogenicity of SARS-CoV-2 cannot be attributed solely to its entry mechanisms or to amino acid substitutions in the spike protein. Remarkably, not all viral genes in our study accumulated mutations during viral evolution. Genes that remained conserved include cofactors of essential RNA replication machinery (NSP7, NSP8, NSP10) and RNA-binding or cap-forming proteins (NSP9, NSP16) (3, 45), In contrast, amino acid substitutions in the viral isolates of our study predominantly affected genes involved in viral replication (NSP4, NSP6, NSP12, NSP13, NSP14), interferon antagonism (NSP3, NSP5, NSP15, ORF3b), host shut-off (NSP1) and autophagy (ORF3a), as well as structural proteins (S, E, M, and N). Consequently, SARS-CoV-2 adaptation to the human host appears to focus on evasion of innate and adaptive immunity, refinement of entry pathways, and optimization of replication efficiency, while core components of the RNA replication machinery remain conserved.

The study by Chen et al. highlighted that amino acid substitutions in NSP6 contribute to a more benign phenotype (22) by promoting more efficient formation of double-membrane vesicles (46) and by reducing pyroptosis (47). In particular, ΔSGF in NSP6 represents an adaptation to replication in humans (48), and, as a gain-of-function change, strengthens the interaction between NSP3 and NSP4 during double-membrane vesicle formation (46). ΔSGF was present in most of our viral isolates and thus does not account for the observed differences between TMPRSS2-dependent and endocytic strains. However, another mutation, M:D3N – prototypical for BA.4/BA.5 lineages (36) – was detected exclusively in the two most endocytic Omicron strains replicating in HEK293T cells (BE.1.1 and BA.5.1). Thus, M:D3N may represent an additional candidate – alongside the spike mutations Δ69/Δ70, L452R, and F486V – that facilitates endocytic entry and warrants further investigation.

Altogether, our study investigated nine SARS-CoV-2 strains isolated throughout the pandemic in four different cell lines. Our data show that viral evolution has broadened the viral entry by shifting the balance toward increased endocytic uptake while preserving TMPRSS2-mediated fusion.

## Limitations of the research

One limitation of our study is that experiments using replication-competent viruses do not allow a clear distinction between effects on viral entry and subsequent intracellular replication. Consequently, the observed effects – particularly in HEK293T cells – may reflect a combination of both processes rather than viral entry alone. A further limitation is that two of the cell lines used (Caco-2, HEK293T) are not of respiratory origin. As such, they may not fully capture the physiological complexity of the *in vivo* situation. In addition, all cell lines employed in this study are immortalized rather than primary cells, which may further limit the direct translational relevance of the findings.

## Data Availability

The datasets presented in this study can be found in online repositories. The names of the repository/repositories and accession number(s) can be found in the article/**Supplementary Material**.
