# Supplementary material for: SARS-CoV-2 evolution enhances endocytic uptake while preserving TMPRSS2-dependent fusion
Source: Front Immunol. 2026 Jan 12;16:1736891. doi: 10.3389/fimmu.2025.1736891 (PMC12832645; doi:10.3389/fimmu.2025.1736891)
Supplement: Supplementary file 1 [file DataSheet1.pdf]

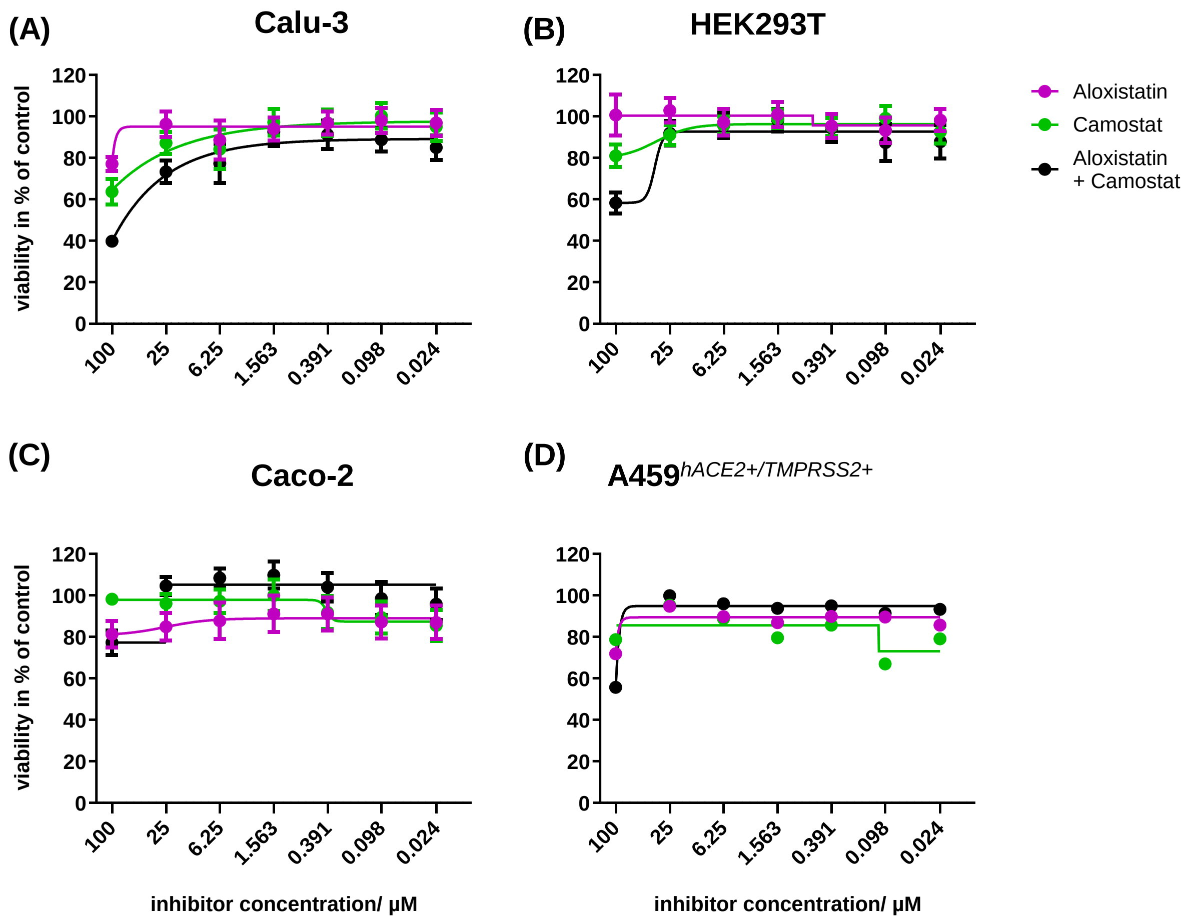

Supplementary Figure S1. Toxicity of SARS-CoV-2 entry inhibitors in four different cell lines. (A) Calu-3, (B) Caco-2, (C) A549$^{hACE2+/TMPRSS2+}$, and (D) HEK293T cells were exposed to SARS-CoV-2 in the presence of increasing concentrations (0.024-100 µM) of aloxistatin, an inhibitor of cathepsin-mediated endocytic entry (pink); camostat, an inhibitor of TMPRSS2-mediated fusion (green); and a 1:1 mixture of both drugs (black) (see Figure 2). Viability was determined at the indicated concentrations at 48h p.i. using the 3-(4,5-dimethylthiazol-2-yl)-2,5-diphenyl-tetrazolium bromide (MTT) assay. Results are expressed as percentage of the viability in uninfected control cells in the absence of inhibitors. Data show mean and standard error of n=11 (Calu-3), n=8 (Caco-2), n=2 (A549$^{hACE2+/TMPRSS2+}$) and n=11 (HEK293T) independent experiments.

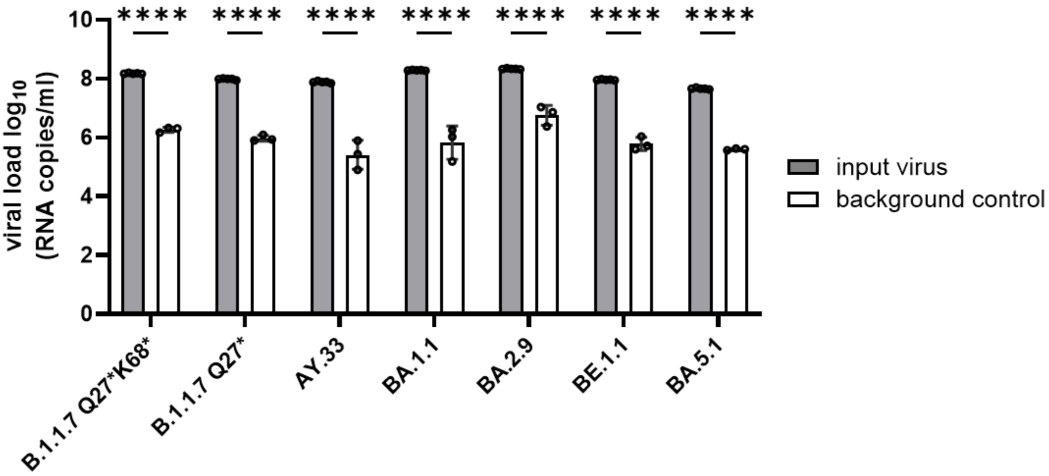

Supplementary Figure S2. SARS-CoV-2 RNA concentrations in the cell culture supernatants before and after removal of the viral inoculum. Viral RNA concentrations were measured in the respective virus stocks at dilutions corresponding to the applied MOI (grey bars). White bars represent background viral RNA levels in PFA-fixed cells after replacement of the inoculum with fresh medium. Data are shown for HEK293T cells and are representative of the other three cell lines. Statistical analysis was performed using two-way ANOVA with Šidák's correction for multiple comparisons.
